# Review of Dissolved Oxygen Detection Technology: From Laboratory Analysis to Online Intelligent Detection

**DOI:** 10.3390/s19183995

**Published:** 2019-09-16

**Authors:** Yaoguang Wei, Yisha Jiao, Dong An, Daoliang Li, Wenshu Li, Qiong Wei

**Affiliations:** 1 College of Information and Electrical Engineering, China Agricultural University, Beijing 100083, China; 2 National Innovation Center for Digital Fishery, Ministry of Agriculture and Rural Affairs, Beijing 100083, China; 3 Beijing Engineering and Technology Research Center for Internet of Things in Agriculture, China Agricultural University, Beijing 100083, China; 4 Precision Agricultural Technology Integration Research Base (Fishery), Ministry of Agriculture and Rural Affairs, Beijing 100083, China

**Keywords:** dissolved oxygen, polarographic, fluorescence quenching, intelligent technologies, sensors

## Abstract

Dissolved oxygen is an important index to evaluate water quality, and its concentration is of great significance in industrial production, environmental monitoring, aquaculture, food production, and other fields. As its change is a continuous dynamic process, the dissolved oxygen concentration needs to be accurately measured in real time. In this paper, the principles, main applications, advantages, and disadvantages of iodometric titration, electrochemical detection, and optical detection, which are commonly used dissolved oxygen detection methods, are systematically analyzed and summarized. The detection mechanisms and materials of electrochemical and optical detection methods are examined and reviewed. Because external environmental factors readily cause interferences in dissolved oxygen detection, the traditional detection methods cannot adequately meet the accuracy, real-time, stability, and other measurement requirements; thus, it is urgent to use intelligent methods to make up for these deficiencies. This paper studies the application of intelligent technology in intelligent signal transfer processing, digital signal processing, and the real-time dynamic adaptive compensation and correction of dissolved oxygen sensors. The combined application of optical detection technology, new fluorescence-sensitive materials, and intelligent technology is the focus of future research on dissolved oxygen sensors.

## 1. Introduction

Dissolved oxygen (DO) refers to free and non-compound oxygen in water or other liquids, which is involved in various biochemical and physiological activities [1,2]. The dissolved oxygen content in water is an important indicator of the water quality and an important factor in water purification. The dissolved oxygen concentration can reflect the self-regulating state of the environment; a high dissolved oxygen content, which is conducive to the degradation of various pollutants in water, indicates that the water can be purified quickly. Conversely, a low dissolved oxygen content leads to the slow degradation of pollutants in water [3]. Dissolved oxygen is critical in many applications [4], such as biomedical fields, food production, and industrial and agricultural production [5,6]. In aquaculture, an appropriate dissolved oxygen content is a prerequisite for the healthy growth of aquatic organisms [7]. For most fish species, if the concentration of dissolved oxygen is lower than 5% [8], the fish are likely to suffocate due to a lack of oxygen [9]. Under high-density fish culture conditions, the dissolved oxygen concentration can change from an optimal level to a lethal level within hours or even minutes, while other key variables in the culture environment are not as dynamic [10]. Therefore, it is of practical significance to study the technology of the real-time and accurate measurement of the dissolved oxygen content [11].

The main determination methods of dissolved oxygen include iodometric titration [12], the electrochemical method, and the optical method [13]. Iodometric titration is a classical laboratory analytical chemical method and an internationally recognized benchmark method for dissolved oxygen determination [14]. The method has a high determination accuracy but has limitations of cumbersome detection procedures and the inability to realize continuous online detection [15]. Electrochemical detection is the most widely used method, within which the polarographic dissolved oxygen sensor is the most common application. This method has a relatively high detection speed, but its detection process consumes oxygen, and sensors based on this principle need to be calibrated and maintained regularly [16]; thus, long-term in situ measurements cannot be realized. The optical dissolved oxygen sensor based on the fluorescence quenching principle is convenient to realize sensor miniaturization [17], does not consume oxygen, and has a high anti-interference ability [18]. Remote acquisition and processing can easily be conducted with the optical fiber used in the optical sensor. In general, the fluorescence quenching sensor requires less frequent maintenance and readily performs continuous online detection, but accurate detection is still difficult to attain [19]. The traditional detection techniques have their own advantages and disadvantages, and the overall accuracy and real-time performance do not meet the actual requirements of different applications.

In actual production and application, the change in the dissolved oxygen concentration is a continuous and dynamic process; thus, a high-accuracy real-time dissolved oxygen detection method is essential. Although many experts want to improve the abovementioned traditional dissolved oxygen detection technology by relying on material science, it is difficult to break through on the whole [20]. An intelligent dissolved oxygen sensor can perform analog and digital signal processing on the collected dissolved oxygen signals, realize intelligent transmission, and achieve a “plug and play” effect. At the same time, through the combination of software and hardware, the intelligent dissolved oxygen sensor can compensate for and correct the detection results in real time [21]. In short, the intelligent dissolved oxygen sensor has the ability of real-time signal acquisition and intelligent data processing, which can meet the requirements of the long-term in situ measurement of the dissolved oxygen content, and effectively overcome the problems of the traditional detection technology [22]. Therefore, it is urgent to develop an intelligent dissolved oxygen sensor with real-time online collection and accurate data processing functions [23].

**Figure 1:**
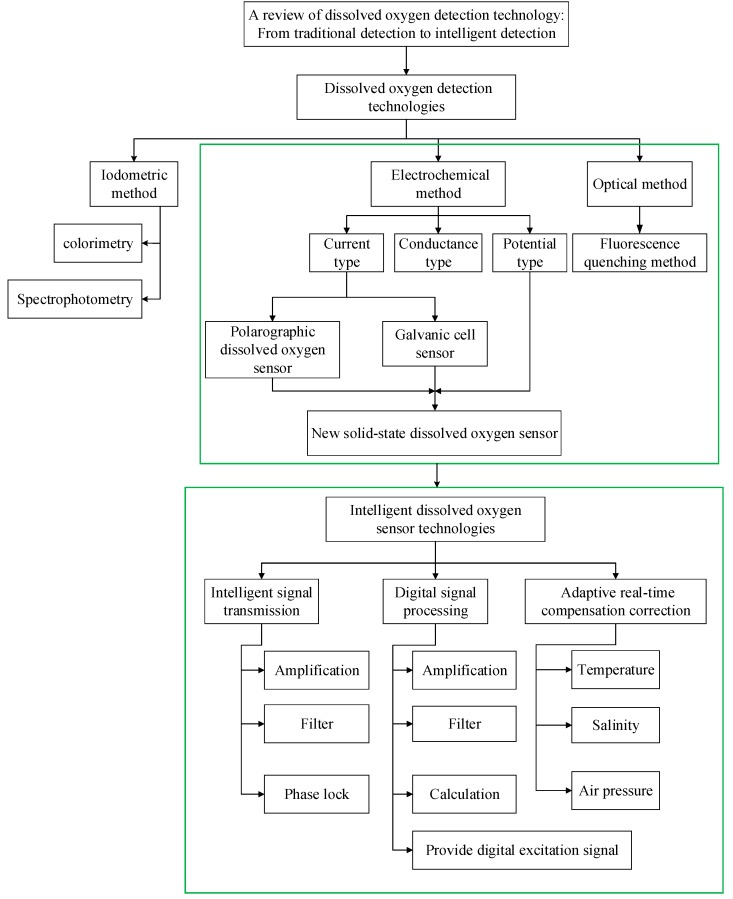
Overview of the article framework.

Wang introduced the principle, materials, advantages and disadvantages, and further development of the Winkler–Clark electrode and fluorescence quenching dissolved oxygen detection method. Although compensation was mentioned, no specific introduction was given and no intelligent concept was introduced [24]. Wang introduced optical oxygen sensors of various principles in a very comprehensive and detailed way, including the development of the new materials used by these sensors, the development of the sensor mode, and their applications in various fields [25]. Unlike those reviews, this review focuses on the intelligence of dissolved oxygen sensors. By comparing the three detection technologies and their improvement methods, it is highlighted that the fluorescence quenching dissolved oxygen sensor based on optical fiber has better performance, but the accuracy and real-time performance still cannot meet the requirements of in situ long-term online measurement; thus, the market is driving the development of optical dissolved oxygen sensor toward an intelligent direction. The full article framework is shown in Figure 1. Firstly, the principle, application, improvement, advantages, and disadvantages of iodine titration, the electrochemical method, and the photochemical method are introduced. Electrochemical and photochemical methods can be used to detect dissolved oxygen online, but they are not suitable for accurate and real-time in situ detection. Therefore, intelligent detection technology of dissolved oxygen is further studied from three aspects: intelligent signal transmission, digital signal processing, and adaptive real-time compensation.

## 2. Dissolved Oxygen Detection Technologies

### 2.1. Determination of Dissolved Oxygen by Iodometric Titration

The iodometric method, also known as volumetric analysis, is the most classical analytical chemical method for the determination of dissolved oxygen and the internationally recognized benchmark method [26]. 

#### 2.1.1. Iodimetry

The principle of iodimetry is to add manganese sulfate and alkaline potassium iodide to water samples to produce manganese hydroxide [13]. Manganese hydroxide is unstable, especially over time, and combines with dissolved oxygen to produce manganic acid. Concentrated sulfuric acid is added to react with the dissolved oxygen (in the form of MnMnO_3_), and potassium iodide is added to the solution to separate the iodine. Starch is used as an indicator, and the iodine released via titration with sodium thiosulfate aids in the calculation of the dissolved oxygen content.

(1) $$4{\mathrm{MnSO}}_{4}+8\mathrm{NaOH}=4\mathrm{Mn}{\left(\mathrm{OH}\right)}_{2}↓\;+\;4{\mathrm{Na}}_{2}{\mathrm{SO}}_{4},$$

(2) $$2\mathrm{Mn}{\left(\mathrm{OH}\right)}_{2}+O_{2}=2H_{2}{\mathrm{MnO}}_{3},$$

(3) $$2H_{2}{\mathrm{MnO}}_{3}+2\mathrm{Mn}{\left(\mathrm{OH}\right)}_{3}=2{\mathrm{MnMnO}}_{3}↓+4H_{2}O,$$

(4) $$4\mathrm{KI}+2H_{2}{\mathrm{SO}}_{4}=4\mathrm{HI}+2K_{2}{\mathrm{SO}}_{4},$$

(5) $$2{\mathrm{MnMnO}}_{3}↓\;+\;4H_{2}{\mathrm{SO}}_{4}+\mathrm{HI}=4{\mathrm{MnSO}}_{4}+2I_{2}+6H_{2}O,$$

(6) $$I_{2}+2{\mathrm{Na}}_{2}S_{2}O_{3}=4\mathrm{NaI}+{\mathrm{Na}}_{2}S_{4}O_{6}.$$

#### 2.1.2. Improvement Methods of Iodimetry

The Winkler method is tedious and time-consuming, and many improvements were made on it in later research. However, the improvement method is mainly to simplify the operation process. Novič achieved partial automation through the flow injection analytical approach [27]. In the improved Winkler method, KIO_3_ was used as the reagent standard to quantitatively determine the concentration of dissolved oxygen. The improved method was fast, with fewer reagents and a sufficient accuracy and precision for daily work. Helm developed an accurate gravimetric micro-Winkler method for the determination of the dissolved oxygen concentration [28]. The method is highly accurate: its expanded uncertainty is in the range of 0.08–0.14 mg∙dm^−3^. Helm implemented the Winkler method with the highest accuracy possible, carefully analyzed the interference from uncertain sources, improved the gravimetric measurement of all solutions, pre-titrated to minimize the effect of iodine volatilization, and accurately detected the end point of the ammeter to calculate the dissolved oxygen in the reagents [29]. In the Winkler method, a large number of samples are often required, and the Winkler method has certain limitations when the sample size is limited [30]. To solve this problem, Shriwastav established a micro-Winkler method for the determination of dissolved oxygen [31]. The sample volume of the improved method for the measurement of dissolved oxygen was 7 mL. If the volume were larger, the overprediction deviation of the dissolved oxygen content could be effectively eliminated. To reduce the difficulty of titration by iodimetry, Li proposed a simple colorimetric method for measuring the dissolved oxygen in 1992 and proposed the application of visual colorimetry [32]. This method has a high adaptability to the rapid measurement of the dissolved oxygen content in fishponds, but the measurement accuracy is poor. Wang used quinone imide cations as color-developing agents to obtain standard color levels for the measurement of the dissolved oxygen in 2002 [33]. Han proposed carbon tetrachloride coloration visual colorimetry in 2011, which can rapidly and stably determine dissolved oxygen, and the detection results are relatively accurate [34]. However, visual colorimetry is only an approximate method, which reduces the advantages of the traditional Winkler method in accurate measurement. Moreover, visual colorimetry does not fundamentally improve upon the operation complexity of the Winkler method and cannot meet the requirements of in situ continuous measurements.

In addition, spectrophotometry is often regarded as a modified method based on iodimetry. Spectrophotometry is a quantitative or qualitative analysis method of a substance based on measuring the light absorption at a specific wavelength or within a certain wavelength range. It was found that the dissolved oxygen in water is fully fixed by manganese sulfate and basic potassium iodide and fully dissolved by concentrated sulfuric acid. The dissolved oxygen content is related to the yellow depth of the solution (the I_2_ content). I_2_ and the excess I^−^ combine to form water-soluble I_3_, and the dissolved oxygen concentration can be determined by measuring the absorption spectrum of the reaction solution [35].

Hu found that 450 nm was the optimal wavelength for the determination of the dissolved oxygen by spectrophotometry, and there was no significant difference between the results and those of iodometry based on a statistical analysis [36]. Labasque et al. reviewed many studies and identified the problem of different measurement values based on wavelength selection [37]. After a comprehensive consideration of the potential factors (temperature, reagent volume, and conservation time) that may change the optimal wavelength, it was found that 466 nm is the optimal wavelength, and it was verified that this method is an effective method for the accurate measurement of dissolved oxygen in water, allowing the measurement of up to 1000 μmol∙kg^−1^ dissolved oxygen. Gao studied the determination of dissolved oxygen by primary and secondary wavelength spectrophotometry [38]. The pretreatment method of iodine sublimation of the samples effectively eliminated the interference from chromaticity, turbidity, and certain substances, with a relative standard deviation of 5% and a minimum detection concentration of 0.05 mg/L. This method is suitable for the analysis of natural water and industrial wastewater. Roland et al. used spectrophotometry to analyze natural water samples collected from lakes and rivers and samples mixed in the laboratory and modified the color and turbidity of water samples [39]. The researchers found that this method can accurately measure the dissolved oxygen in a wide concentration range (4–13 mg/L) and can distinguish a change in dissolved oxygen of 0.05 mg/L. This method is faster and simpler than the iodometric method, but it is not suitable for the measurement of dissolved oxygen at low concentrations. Zhang investigated the iodine-mediated etching of gold nanorods (Au NRs) in the presence of iodate. The short wavelength transformed by this method can be detected by spectrophotometry to obtain the content of dissolved oxygen. This method is simpler and more accurate than visual colorimetry and has higher applicability in the detection of dissolved oxygen. [40]. Spectrophotometry based on the flow injection analysis method simplifies the determination of the dissolved oxygen. Pai et al. designed an air-free pipette system that combines wide-hole flow tubes with a conventional spectrophotometer [41]. This method improves the colorimetric method of iodine separation and avoids the error of iodine volatilization loss caused by the contact with air and the error introduced by the manual calibration test tube. The relative deviation of measurement is no more than 0.5%, with high precision and simple operation. Sakaia et al. assembled a five-channel flow system with a newly designed 16-way valve, thereby realizing the automatic online detection of the dissolved oxygen concentration [42].

The speed of spectrophotometry is much higher than that of iodimetry, and spectrophotometry also does not require repeated calibrations. The operation is greatly simplified, and the error caused by the volatilization of iodine is reduced. Spectrophotometry is widely used in laboratory data analysis because it is difficult to measure the dissolved oxygen online in real time. At the same time, the spectral absorption difference between free iodine and complex iodine will lead to errors in the results, which limits its application to a certain extent.

In summary, neither iodometric titration nor its improvement method fundamentally solved the complex operation problem. Although the Winkler method has a high accuracy, it also has certain limitations; during the detection process, the oxygen in the water is consumed, and the titration reagent pollutes the environment. In addition, the color and turbidity of water samples cause errors in the measurement results. When water contains substances such as iodate [43], hydrogen peroxide, or nitrite [44,45], these compounds interfere with the determination of the dissolved oxygen [46]. Because the titration process is complex and tedious, the Winkler method can only be used in a laboratory environment, and it is difficult to realize in situ continuous online measurements.

### 2.2. Electrochemical Dissolved Oxygen Sensor

Electrochemical detection is a common measurement method of dissolved oxygen [47]. Electrochemical dissolved oxygen sensors are now the most widely used sensors because they can realize in situ online measurements [48]. Most of the research on electrochemical dissolved oxygen sensors focuses on improving the sensitive materials, and it is expected that the service life of the sensor will be improved by using new sensitive materials [49]. Electrochemical dissolved oxygen sensors can be divided into current-type, conductance-type, and potentiometric-type sensors according to their different output signals. The current-type sensors are divided into polarographic and galvanic types. The conductance-type sensor is based on the conductivity change of the electrolyte solution after oxidation or reduction. Since the electrode potential has a certain relationship with the concentration of the measured component, the potentiometric-type sensor senses the change in the dissolved oxygen concentration through the change in the electrode potential [50].

#### 2.2.1. Polarographic Dissolved Oxygen Sensor

**Figure 2:**
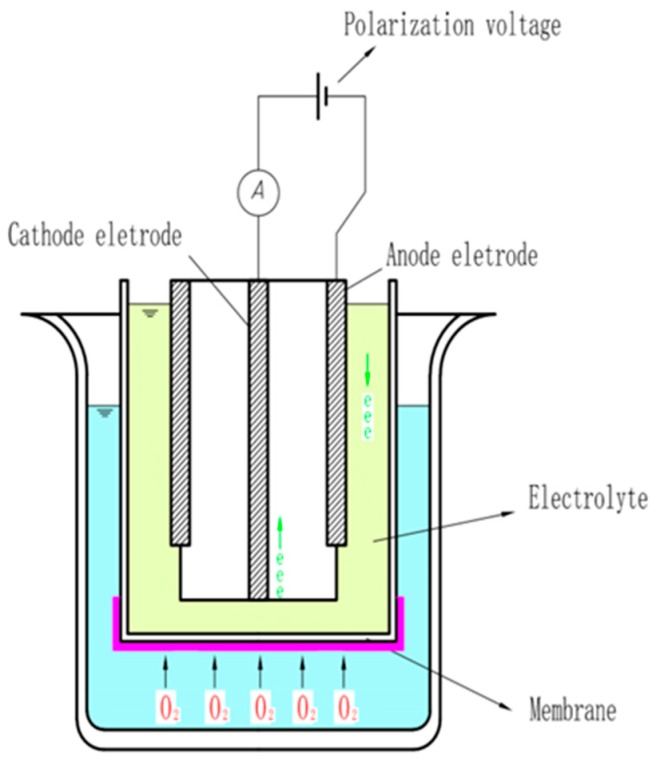
Structure diagram of the polarographic dissolved oxygen sensor probe.

Polarography is a method for determining the concentration of substances in solution by measuring the current–potential (or potential–time) curve of polarized electrodes during electrolysis. Initially, the polarographic sensor only used a pair of bare electrodes to measure the dissolved oxygen, and the electrode was immersed directly in the solution; thus, the surface of the electrode was easily contaminated by impurities in the solution to be tested. Subsequently, Clark proposed a film-coated electrode wrapped with a polymer plastic film and improved the structure of the polarographic electrode. As shown in Figure 2, the probe of the current polarographic dissolved oxygen sensor is mainly composed of working and auxiliary electrodes, an air-permeable film, and an intracellular electrolyte, and wires are added between the two electrodes to form a loop. When a voltage is applied between the working and the auxiliary electrodes, oxygen molecules are reduced on the working electrode, allowing more oxygen atoms to pass through the selective air-permeable film, thus forming a diffusion current. Polarographic sensors are widely used because they overcome the problem that the electrode is easily polluted in the early stage. The specific reaction process of the working and auxiliary electrodes is expressed by the following chemical equations:

(7) $$O_{2}+2H_{2}O+4e\;\to 4{\mathrm{OH}}^{-},$$

(8) 4Ag+4Cl →4AgCl+4e.

The oxidation and reduction of dissolved oxygen by the polarographic electrodes is a slow kinetic process requiring a high potential; thus, the electrocatalytic reaction of oxygen attracted much attention. Researchers used a variety of electrocatalytic materials to improve the electron transfer efficiency between the dissolved oxygen and the electrode surface [51,52]. In 2006, Lin analyzed the characteristics of vitamin B_12_ and used it as the modification material of the electrode to improve the electrocatalytic activity. The experimental results showed that the electrocatalytic effect of the sensor was good, not only improving the response time but also reducing the maintenance frequency [53]. In 2010, Damos constructed a supramolecular membrane with high stability by using cyclodextrin molecules, which promoted the reduction/oxidation (redox) reaction between the electrodes and dissolved oxygen. This sensor can shorten the polarization time of the electrode under low voltage [54]. In 2012, Martin explained the kinetic mechanism of the oxygen reduction and also enhanced the circuit current by coating the electrode surface with a film made of a new material [55]. In 2013, considering the cost, Hsu decided to use hemin as a substitute for the expensive electrocatalytic material and chose a silicone rubber permeable membrane to prolong the service life of the sensor. The research results showed that the sensor had a high catalytic activity and strong anti-pollution ability [56]. Based on the excellent electrochemical properties of boron-doped diamond, in 2014, Sliva described a new type of microelectrode sensor, which is coated with modified materials on the electrode surface. The response speed of the sensor is significantly improved, and the measurement can be stable for a period of time [57]. Xin used an all-new tri-electrode balanced structure sensor, which can not only greatly shorten the polarization time but also effectively extend the service life [58]. In 2015, based on the electrical displacement, Fu synthesized a reduced graphene silver oxide NP (RGO/Ag) composite-modified straw carbon electrode (GCE) [59]. The proposed dissolved oxygen sensor has good repeatability, reproducibility, and anti-interference ability. In 2017, considering not only the excellent electrochemical properties of carbon but also the shape and size of the sensor electrode, Clark designed a carbon-fiber micro-column electrode that showed sensitivity to dissolved oxygen [60]. This technique not only performs well under ideal experimental conditions, but was also proven to be effective in the detection of dissolved oxygen in different aqueous solutions. In 2018, Wiranto used thick-film technology to make a sensor based on an RuO_2_ working electrode that has high activity and can produce a sensitive reaction to dissolved oxygen in five minutes. The overall design and performance provide the possibility of the online detection of the dissolved oxygen concentration [61]. A comparison of several polarographic dissolved oxygen sensors is shown in Table 1.

In the process of exploring sensitive sensor materials, the electrochemiluminescence (ECL) principle of the sensor was discovered [63]. Little attention was paid to the ECL characteristics of TiO_2_ before 2008. Based on these characteristics, Lin further studied the linear relationship between ECL and the dissolved oxygen concentration. Then, he designed a dissolved oxygen sensor that is sensitive and easy to prepare. Ye et al. summarized the reports on ECL behavior and found that very little was determined about the ECL behavior of luminol on a TiO_2_-modified electrode. Therefore, they studied a new modified electrode and compared the detection deviations of the two ECL peaks and concluded that ECL-2 had a better performance [64]. Zheng et al. used an Ru(bpy)_3_^2+^/Nafion film-modified glassy carbon electrode (GCE) for an ECL experiment. O_2_ participates in the reaction process and can react with the electrode to produce a strong ECL phenomenon. The experiment proved the sensitivity of O_2_ to the improved membrane [65]. ECL sensors have the advantages of a high sensitivity, good selectivity, good repeatability, and ease of control, which promote the wider market application of electrochemical dissolved oxygen sensors [66].

Despite the rapid development of polarographic electrochemical dissolved oxygen sensors that led to great breakthroughs in materials and other aspects, there are still some problems and deficiencies. Although polarographic sensors can realize continuous dissolved oxygen measurements, their measurement process consumes oxygen and cannot meet the requirements of real-time measurements in still or low-velocity liquids [67]; thus, these sensors produce large errors in low-velocity application scenarios such as ponds. The measurement accuracy and response time of polarographic sensors are also severely constrained by the diffusion coefficient of the permeable film, resulting in a low accuracy, poor stability, and high susceptibility to interferences. To overcome the impossibility of accurate measurement caused by the water flow velocity and permeable film, Luo et al. explored the relationship between the liquid velocity and the measurement of the dissolved oxygen concentration and found that the current increases with the velocity until it reaches saturation. Based on this, they described a microfluidic Clark-type oxygen sensor based on a low-temperature cofired ceramic (LTCC), demonstrating the effect of the liquid flow rate on electrochemical sensors. The sensor is not complex to manufacture and is easy to use [68]. Janzen redesigned the polarographic sensor and developed the SBE 43 dissolved oxygen sensor. The sensor keeps the internal battery energized to achieve the effect of continuous polarization, reducing the time of pre-polarization each time so that the sensor can respond quickly. The SBE 43 dissolved oxygen sensor adopts a unique plumbing arrangement to form an aerobic state around the sensor, which also promotes the reaction between the dissolved oxygen and electrode to some extent [69]. The SBE43, developed by Sea-Bird, was in commercial use for a long time, and the sensor is now very mature and often used in a variety of applications.

In addition to the requirement of the liquid velocity, the dissolved oxygen sensor also attaches great importance to real-time performance when the concentration of dissolved oxygen needs to be measured in aquaculture and similar scenarios. Because polarographic sensors need to be polarized before use, the traditional polarization process takes a long time, and it is difficult to meet the real-time requirements. Against this background, Huang proposed a parallel energy storage capacitor to the precision resistor in the current–voltage conversion circuit. Before energizing the measuring system, the sensor undergoes a low-voltage pre-polarization process, which can greatly reduce the polarization time [70]. The technique was developed by the team of authors, and is not limited to theoretical research. Other studies changed the conventional steady-state mass transfer process into a transient unsteady pulse mass transfer process [71], in which a pulse is used to motivate the electrode to realize polarization [72]. This impulse excitation has clear advantages; the system is less dependent on agitation (sample flow rate). The diffusion current is much higher than that due to direct current (DC) polarization, and the sensitivity of the electrode is enhanced. The consumption of the electrolyte is greatly reduced, and the electrode life is prolonged [73]. Moreover, the unsteady mass transfer process inhibits the attachment of impurities to the electrode in water.

Although polarographic sensors have many measurement limitations, these sensors are widely used in detection in natural water and sewage due to the simple structure, long probe life, wide range (especially in the ppb-scale dissolved oxygen measurement cases), high accuracy, relatively mature technology, and the ability to achieve the online detection of dissolved oxygen [74]. From theoretical research to practical manufacturing, the performance of the sensor is gradually narrowing the gap with commercial products. At present, research on polarographic dissolved oxygen sensors mainly focuses on the improvement of the electrode and permeable film materials, and a novel electrochemiluminescence sensor was established, which obtains sensor measurement results that have a better stability and accuracy. At the same time, by changing the polarization mode, the polarization voltage of the sensor is improved as the pulse voltage of the unsteady mass transfer process, which reduces the electrode polarization time and overcomes the influence of electrode polarization on the detection accuracy to a certain extent.

#### 2.2.2. Other Electrochemical Dissolved Oxygen Sensors

Other commonly used electrochemical dissolved oxygen sensors include the galvanic cell-type, conductance-type, and potentiometric-type sensors. The structures of the galvanic cell-type and polarographic sensors are similar [75], and a reduction reaction occurs when oxygen molecules in the water sample reach the cathode, but no additional polarization voltage is needed [76]. According to the principle of the galvanic-type dissolved oxygen sensor, Han-Peng prepared a galvanic-type oxygen sensor by using an active copper electrode as the anode, a gold electrode as the cathode, and a KOH solution as the electrolyte [77]. Considering the cost and other limitations of traditional methods [78], biofuel cells are also used for the measurement of dissolved oxygen. Zhang successfully developed a simple and accurate submersible microbial fuel cell (SBMFC) sensor that can rapidly monitor the dissolved oxygen level in various environmental waters. The reported results demonstrated reproducible microbial-mediated current generation as a function of the dissolved oxygen concentration [79]. Table 2 compares the polarographic and galvanic electrochemical dissolved oxygen sensors.

The conductive dissolved oxygen sensor uses thallium or other compounds to react with the oxygen molecules in water to generate thallium ions. Because the chemical reaction on which the sensor is based is specific to oxygen molecules, the concentration of dissolved oxygen can be calculated by measuring the changes in the conductivity of water samples. Yu used a standard solution of hydrochloric acid to titrate the reaction product of thallium and dissolved oxygen in water, and the end point of the titration was indicated by the conductance method [80]. Zhang submitted a new method based on the conductance measurement method. A small amount of water was drawn from the pipe by a precision water pump into the conductance measurement pool, and thallium metal was used as the consumable material for indirect measurement, which did not pollute the water body [81].

The potentiometric dissolved oxygen sensor contains an oxygen-sensitive material fixed on the surface of the working electrode [82]. When oxygen molecules are close to the sensitive surface, the working electrode is polarized, and the dissolved oxygen potential can be obtained by measuring the voltage difference between the working electrode and the reference electrode [83]. This potential value is directly proportional to the logarithm of the concentration of dissolved oxygen in the water sample; hence, the dissolved oxygen concentration value can be determined. Martínez-Máñez, using RuO_2_ as the active material and TiO_2_ or polyisoftalamide diphenylsulfone (PIDS) as the membrane, developed a potentiometric dissolved oxygen sensor with thick-membrane technology [84]. Zhang proposed a new method, the quasi-equilibrium method. In this method, a platinum electrode was polarized at the anode and cathode, which improved the problem of a long electrode balance time [85]. Peter realized dissolved oxygen detection through a combination of conventional potentiometry with active pre-polarization protocols, with a sensitivity within the range of 60 mV/dec oxygen concentration [86].

Dissolved oxygen sensors, such as galvanic cell-type, conductance-type, and potentiometric-type sensors, are widely used in monitoring dissolved oxygen in water quality because of their simple structure and easy online measurement. 

#### 2.2.3. New Solid-State Dissolved Oxygen Sensor

The traditional electrochemical sensors mostly use an electrolyte solution as the conductive medium, which requires a higher electrode structure and performance. The liquid electrolyte between the selective breathable film and the electrode increases the manufacturing difficulty of the sensor and reduces the durability of the sensor, making it difficult to preserve the sensor for a long time [87]. Electricity is easily affected by liquid leakage, and its stability and reliability are poor; in addition, it is difficult to realize miniaturization. With the development of chemical technology, experts in related fields proposed using hydrogels, solid electrolytes, or solid proton-conductive materials to replace the liquid built-in electrolyte and simultaneously used micromechanical processing technology to realize the miniaturization of sensor electrodes. Its main advantages are the ease of making ultrathin electrodes and the ease of changing the shape without the need for metal shell packaging, which fundamentally solves the problem of leakage; thus, the safety, reliability, and miniaturization were greatly improved [88]. The use of solid electrolyte prolongs the life of dissolved oxygen sensor and improves the shortcomings of traditional liquid electrolyte sensor [89]. It can be used in many applications, has a broad space for development, and will become the future research hotspot.

Based on the above technical background, researchers developed an all-solid dissolved oxygen sensor, which to some extent overcomes the limitations of sensor size and maintenance frequency. Glasspool considered the use of screen-printing technology to encapsulate the electrode and solid electrolyte gel in the cavity when designing the sensor. Such a design is easy to miniaturize and can prevent the poisoning of the cavity electrode [90]. Wang et al. prepared a sensor by overlaying platinum thin-film electrodes with a solid-state proton conductive matrix (PCM) coating and used the spin coating process to cover the surface of the planar membrane electrode with a solid proton-conductive material instead of the built-in electrolyte in the traditional Clark dissolved oxygen sensor [91]. The sensor was shown to have a suitable performance in terms of the long-term stability, reliability, hysteresis, linearity, and sensitivity. Lee et al. proposed an electrochemical dissolved oxygen sensor with three electrodes, which are covered by a solid electrolyte and do not require an oxygen-permeable membrane [92]. The dissolved oxygen sensor can be used for real-time and continuous measurements, which solves the problem of the membrane degradation of traditional electrochemical sensors.

New solid-state electrochemical sensors adopt a solid electrolyte material, which makes the structure relatively easy to miniaturize. At the same time, the solid electrolyte material is stable and reliable, which earned it increasing attention in the research on electrochemical dissolved oxygen sensors.

### 2.3. Optical Dissolved Oxygen Sensor

In recent years, oxygen sensors with different optical principles developed rapidly, such as the fluorescence quenching principle, the phosphorescence quenching principle [93,94,95], the near-infrared principle, and the absorption principle [96,97]. At present, most optical dissolved oxygen sensors are based on fluorescence quenching principle. Dissolved oxygen detection by fluorescence quenching was firstly confirmed in 1939, and the dissolved oxygen sensor based on the fluorescence quenching principle is now commercially available [98]. The aforementioned dissolved oxygen sensor is a device for determining the dissolved oxygen concentration by means of quenching the fluorescence substance after collision with dissolved oxygen molecules [99]. After the fluorescent substance absorbs visible or ultraviolet light of a specific wavelength, its electrons gain energy and become excited and release energy to return to the ground state by emitting fluorescence. Since the collisions between oxygen molecules and excited fluorescent substances interfere with the excitation process of fluorescent substances, the content of oxygen molecules in the water samples can be determined according to the fluorescence intensity or the fluorescence lifetime generated at the sensitive interface.

**Figure 3:**
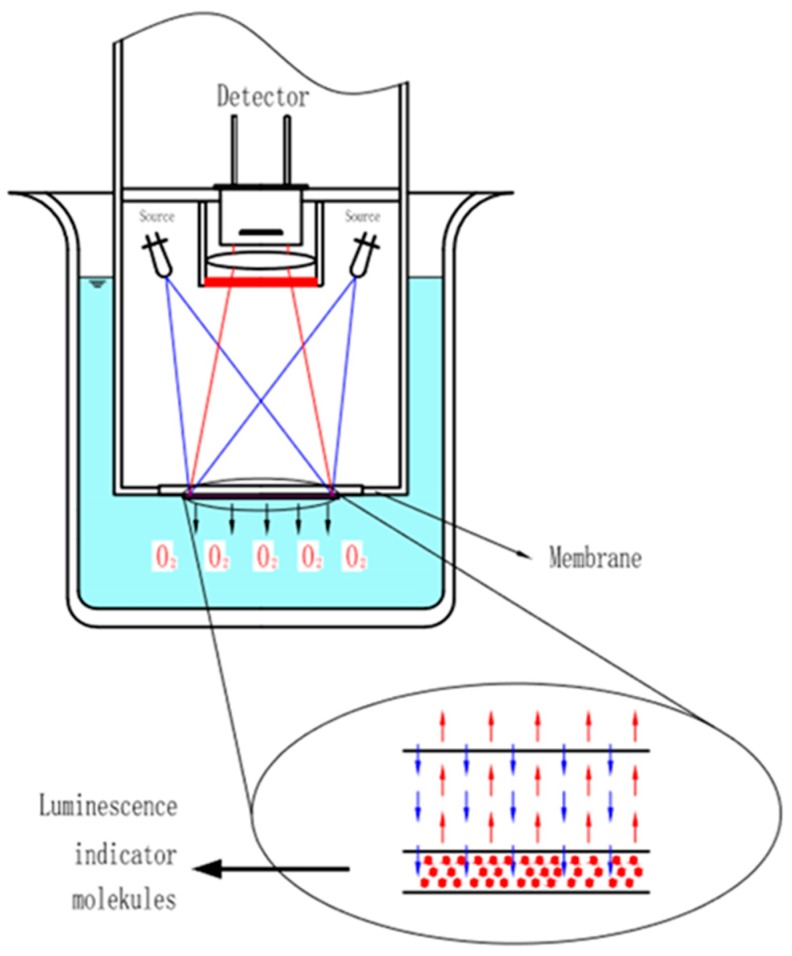
Fluorescence quenching of dissolved oxygen sensor probe.

As shown in Figure 3, the dissolved oxygen sensor based on the fluorescence quenching principle is composed of excitation light sources, a substrate film attached to fluorescence-sensitive substances, and an optoelectronic detection element. The fluorescence quenching reaction occurs at the interface of the sensitive film when the fluorescence sensor is put into water and stimulated by the light source.

The principle of fluorescence quenching follows the Stern–Volmer equation [100].

(9) $$\frac{I}{I_{0}}=\frac{\tau }{\tau_{0}}=1+K_{SV}\left[O_{2}\right],$$

where *I*_0_ and $\tau_{0}$ are, respectively, the fluorescence intensity in anaerobic conditions in the solution and the fluorescence lifetime, and I and τ are an arbitrary fluorescence intensity of a different dissolved oxygen concentration and its fluorescence lifetime. In addition, K_SV_ refers to the fluorescence quenching constant, and O_2_ is the measurement of the oxygen concentration. It can be seen from the equation that, for the dissolved oxygen sensor based on the fluorescence quenching principle, the concentration of dissolved oxygen is linearly correlated with the fluorescence intensity and fluorescence life [24]. When considering the calculation of the fluorescence intensity [101], sensors usually measure the fluorescence intensity by monitoring the emission intensity of oxygen-sensing materials at various oxygen concentrations [102]. Zhao developed an intelligent optical sensor based on fluorescence intensity detection that is composed of a light-emitting diode (LED) and photodiode and does not need an optical fiber [103]. The sensor is portable and exhibits good linearity. Dissolved oxygen sensors based on the luminescence intensity are sensitive to interference from factors such as the power drift of the light source, the turbidity of the sample, the detection background, and the photobleaching of the fluorescent dye itself [104]. Therefore, it is difficult to construct a stable and reproducible dissolved oxygen sensor based on the fluorescence intensity. The fluorescence lifetime is an intrinsic parameter of the fluorescence signal and independent of the performance of external stray light and photoelectric device [105]. The measurement of the fluorescence lifetime can solve the fluorescence intensity problem to a certain extent [106].

In terms of sensitive film elements, fluorescence quenching dissolved oxygen sensors can improve the sensor performance by selecting different fluorescence materials and film substrate materials. Common fluorescent substances include pyrene, pyrene butyric acid, and fluoranthene, as well as other polycyclic aromatic compounds [107]. Such fluorescence-sensitive substances do not consume dissolved oxygen, have a high response rate (less than 50 ms), and are relatively stable. To improve the detection sensitivity, ruthenium–chromium complexes, as well as platinum phosphor porphyrins such as platinum tetrafluorophenyl porphyrins (PtTFPP) and platinum octaethyl porphyrins (PtOEP), are also widely used as fluorescence indicators in optical oxygen sensors to detect the concentration of dissolved oxygen [108,109]. The fluorescence intensity of these types of metal compounds corresponds to the dissolved oxygen partial pressure, and the atomic excited state of these metal compounds has a long life and is very stable, making these compounds ideal fluorescence sensitivity indicators. Chen briefly summarized the common matrix materials used in various studies and proposed a type of dissolved oxygen sensor coated with a Pt(II) complex medical polymer film. The sensor used a cylindrical core waveguide structure, which showed the characteristics of rapidity and a low signal-to-noise ratio (SNR) when measuring the dissolved oxygen in a large dynamic range [110]. The technique is still in the laboratory and will be explored in the future to see if it can be used in liquid phase for long periods of time. In addition, environmental concerns led researchers to focus on the use of green materials. Silva et al. extracted chlorophyll A from cabbage and combined it with zinc to form a complex, which was used in an optical sensor with high sensitivity, and the *R^2^* of fluorescence intensity and voltage inhibition reached 0.9809 [111]. The principle of optical oxygen sensors is the oxygen-quenching effect of the fluorescence of the luminescent molecules (luminescent body) fixed in the matrix; thus, the choice of substrate material is also an important factor impacting the performance of the sensor. At present, many materials are used as matrixes, including silicone rubber [112], silica gel [113], sol–gels, and polymers [114,115], most of which have a high oxygen permeability, good mechanical and chemical stability, and excellent optical transparency [116,117,118,119,120]. Peng proposed a new method for the determination of dissolved oxygen using a porous optical fiber [121]. Sensitive films containing ruthenium-bipyridine ([Ru(DPP)_3_]Cl_2_) were modified on the inner pore walls of the porous fiber using sol–gel film-forming technology, and the test probe was then prepared. The measurement sensitivity of the experiments was 3.6 ppm, the sensor response curve measured in the range of 0–20 mg/L was approximately linear, and the sensor had a quick response time of 200 ms. Noor et al. utilized sol–gel technology to dip and coat oxygen indicators on the distal end of the optical fiber, and the designed sensor obtained consistent sensitivity results and a reliable drift performance [122].

In terms of the signal transmission components, an optical fiber has the advantages of a small size [123], low weight, good electrical insulation, suitable safety, anti-electromagnetic interference ability, and high sensitivity; it is also easy to use existing optical communication technology to form a telemetry network [124], leading to optical fibers becoming the first choice of information transmission components. In the detection method of dissolved oxygen, the optical fiber is very suitable for the transmission and detection of fluorescence [25]. Most of the reported fluorescence quenching dissolved oxygen sensors use an optical fiber to transmit fluorescence signals [125]. Fiber-optic dissolved oxygen sensors usually use the sol–gel method to coat fluorescence indicators on the surface of the fiber-optic probe [126], and the fluorescence signal after oxygen-molecule quenching is transmitted to the photoelectric converter through the fiber [127]. Then, the corresponding dissolved oxygen concentration value can be obtained after signal processing. Zhao prepared a plastic fiber-optic sensor based on the fluorescence quenching principle [128]. The experimental results showed that the relative fluorescence intensity of the sensor had a linear relationship with the concentration of dissolved oxygen, and the fitting coefficient was up to 0.9807. Moreover, the sensor had good reversibility. Mahmud prepared a type of fiber-optic oxygen sensor to evaluate its performance in gas and liquid phases [129]. According to the experimental results, the optical dissolved oxygen sensor showed a high sensitivity and stability in the water environment. In the field of fiber-optic dissolved oxygen sensors, past work reported only the use of short fibers. However, Mahmud examined long glass fibers up to 20 m long, whereby the electronics could be located further away from sensor elements [130]. This sensor has more flexibility when deployed and is important for the online detection of dissolved oxygen. Table 3 shows a comparison of several fluorescence quenching dissolved oxygen sensors.

On the basis of using an optical-fiber transmission medium, researchers improved the sensor performance by optimizing its structure. The single-wavelength fluorescence intensity detection mode is easily affected by the environment and the instrument itself, and the ratio fluorescence intensity detection method based on double emission can effectively make up for the deficiency of single-wavelength fluorescence detection [131]; thus, the ratio fluorescence sensor became a current research hotspot of oxygen sensors. Ratio-type optical oxygen sensors refer to a type of sensor in which two or more fluorescence emitters are fixed in the same sensing matrix. Different fluorescence emitters have different responses to oxygen, and they emit light in different bands. Zang did not simply analyze the fluorescence quenching characteristics but further considered the dual spectral characteristics of phosphorescence and fluorescence, balancing the two to produce a strong light emission effect. The sensor designed based on this principle can obtain accurate detection results for the dissolved oxygen concentration [132]. Chu produced a ratio-type fiber-optic dissolved oxygen sensor, including a plastic fiber coating at one end with Pd(II)/CdSe quantum dots (QDs) or Pt(II)/CdSe QDs embedded in the sol–gel matrix, which can effectively suppress the effects of spurious intensity fluctuations of the excitation source and the optical transmission characteristics of the fiber [133]. Zike developed a ratio-type dissolved oxygen sensor [134]. Ru(DPP)_3_Cl_2_ and coumarin 6 were combined in a poly(methyl methacrylate) (PMMA) matrix doped with silver NPs (Ag NPs) to prepare a ratio-type oxygen sensor film. The results showed that the sensor has a good stability, response time, and light stability. Xu selected oxygen-sensitive Eu^3+^ and oxygen-insensitive ligand materials for making ratio sensors. Compared with the simple fluorescence sensor, this sensor can improve the detection speed, accuracy, and service life [135]. Li realized the advantages of a fluorescence dissolved oxygen sensor and designed a sensor based on the fluorescence quenching principle [136]. A fluorescence sensor cap replaced the electrolyte and permeable film in the traditional electrochemical method, which was convenient for the replacement and maintenance of the sensor film, and improved the sensor’s ability to resist the interference from sulfides and other substances. The sensor provides a real-time and accurate evaluation basis for water quality evaluation.

Miniaturization is an inevitable trend in the development of sensors [137]. It is difficult to produce accurate microscale oxygen sensors using traditional electrochemical measurement methods. The introduction and application of optical fibers promotes the convenient design of miniaturized dissolved oxygen sensors. Therefore, the fiber-optic dissolved oxygen sensor based on the fluorescence quenching principle meets the needs of the miniaturization of the sensor more easily [138]. In recent years, the development of optical sensors based on waveguide sensing attracted much attention due to their analytical potential and the possibility of miniaturization. Xiong coated the fiber surface with an organically modified silicate (ORMOSIL) film with immobilized [Ru(bpy)_3_]^2+^ fluorophores [139]. When light was propagated by total internal reflection (TIR) in the fiber core, evanescent waves generated by the fiber surface could stimulate the fluorescence emission of the fluorophores, thus enhancing the fluorescence quenching reaction. This design simplifies the manufacturing process and successfully miniaturizes the sensor, making the sensor readily portable and less costly, achieving a higher detection sensitivity at low dissolved oxygen concentrations. Chang-Yen et al. combined an oxygen-sensitive fluorescent dye with microfabricated Cytop/spl trade/waveguides and integrated the single chip into the sensing substrate to miniaturize the optical oxygen sensor [140]. This design was applied to the measurement of the biological dissolved oxygen with strict requirements, which also has important reference significance for the measurement of the dissolved oxygen in industrial and agricultural environments. Yang et al. designed an internal fiber sensing structure suitable for microfluidic devices [141]. Special capillary fiber and ring waveguides were used to sample the surface of the fiber and calculate the luminescence intensity to obtain the dissolved oxygen concentration. This design has a good linear Stern–Volmer relationship in the full concentration range of oxygen and a short response time.

A comparison of three dissolved oxygen detection methods is shown in Table 4. The advantages of fluorescence quenching optical dissolved oxygen sensors are very clear [142]. This type of sensor overcomes the disadvantages of traditional polarographic dissolved oxygen sensors, such as poor stability and reliability, strong dependence on the fluid velocity, and easily influenced electrode polarization [143], and has a higher sensitivity, faster response, zero oxygen consumption [144], low weight, high precision [145], and excellent electromagnetic interference (EMI) resistance, using the advantages of a good effect in harsh environments [130]. For example, Janzen improved the electrochemical dissolved oxygen sensor SBE 43 [69], but the sensor drift is very serious, mainly due to the easy fouling; thus, it needs frequent maintenance and calibration [146,147]. To solve this problem, Seabird launched the SBE 63 dissolved oxygen sensor, which adopts the fluorescence quenching method and has good accuracy and real-time performance. In particular, the sensor has a long life and is more robust, and it is becoming a better market choice to replace the SBE 43 dissolved oxygen sensor. Therefore, optical dissolved oxygen sensors based on fluorescence quenching are widely used in industrial and agricultural production.

The common dissolved oxygen detection methods include iodometric titration and electrochemical and optical methods. Iodometric titration is a laboratory method with high accuracy, but the operation process is very complicated, and the process is easily disturbed by turbidity, nitrite, iron ions, free chlorine, and other substances in the water samples. Although there are many improved methods of iodometric titration, such as visual colorimetry, these methods are yet to fundamentally overcome the shortcomings of the iodometric method, and it is difficult to achieve the goal of in situ online measurement. The main electrochemical sensors are polarographic, galvanic cell-type, conductance-type, and potentiometric sensors. Among these sensors, polarographic sensors are the most widely used, and they easily realize online measurements. However, due to the water flow rate limitation, the measurement process consumes dissolved oxygen, which leads to corrosion, and the method has poor stability and reliability and requires regular maintenance. To solve the miniaturization problem of the electrochemical dissolved oxygen sensor, a new completely solid-state sensor was studied. The dissolved oxygen sensor based on the fluorescence quenching principle achieved much in the research on sensitive materials and matrix materials, and its effect is good. The approach has the advantages of a high anti-interference ability, precision, stability, and reliability. In addition, the fluorescent dissolved oxygen sensor uses an optical fiber as the medium to transmit the sensitive information measured. The sensor relies on an optical fiber to obtain optical measurements, and the sensor can easily be miniaturized and has a high anti-interference ability; the sensor conveniently realizes real-time online measurement, as well as signal transmission and processing [144]. The fluorescent dissolved oxygen sensor, thus, has broad prospects for development [148].

## 3. Intelligent Dissolved Oxygen Sensor Technologies

Intelligent sensor technology refers to the intelligent transmission, digital information processing, compensation, and correction of signals detected by sensors, which improves the accuracy, stability, reliability, and anti-interference ability of the sensor detection [149,150]. The traditional sensor measurement has the disadvantages of a poor stability, large drift, short service life, poor anti-interference ability, long response time, and low accuracy [151], which requires users to regularly conduct calibration and maintenance; thus, the traditional sensor measurement hardly meets the requirements of long-term in situ measurement. To solve these problems, there is an urgent need to develop intelligent sensors with the functions of intelligent signal transmission, digital signal processing, field-adaptive compensation and correction, etc. [22,152]. An intelligent sensor is a type of sensor with a microprocessor that has functions of information detection, information processing, information storage, logical thinking and judgment, etc. [153]. The Institute of Electrical and Electronics Engineers (IEEE) developed a series of standards related to smart sensors [154].

**Figure 4:**
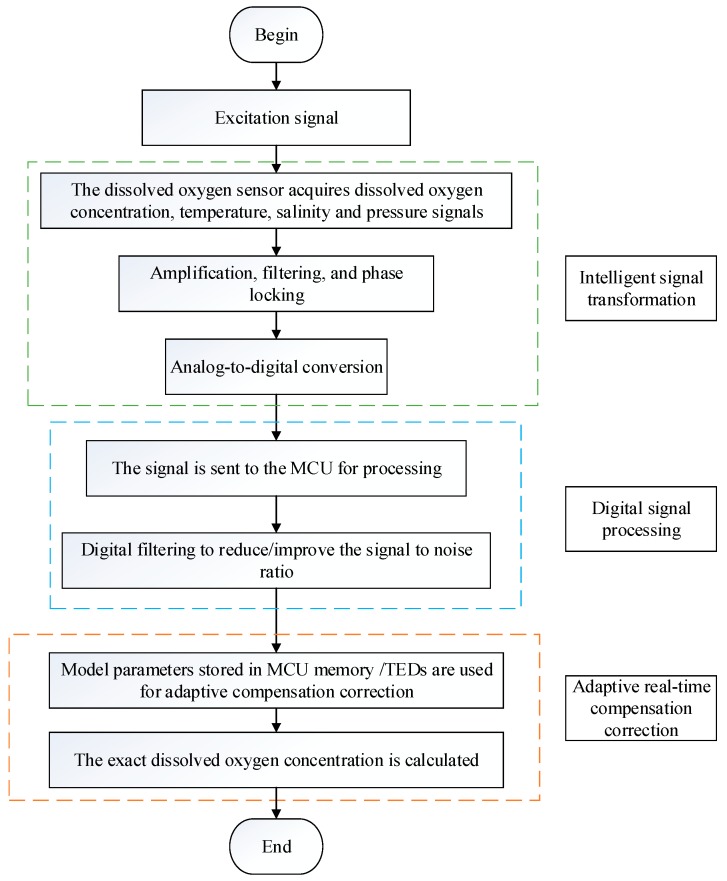
The signal processing flow of the intelligent dissolved oxygen sensor.

Traditional dissolved oxygen sensors cannot easily achieve the in situ real-time rapid detection of the dissolved oxygen concentration; that is, the accuracy of the detection results is not high and cannot meet the real-time requirements. According to the specific requirements of the intelligent sensor, a new type of intelligent dissolved oxygen sensor is also needed in the detection of dissolved oxygen. Figure 4 shows the signal processing flow of the intelligent dissolved oxygen sensor. Through analog signal processing technology, the collected signal is intelligently transferred to the microprocessor, and then the corresponding dissolved oxygen value is calculated by digital signal processing technology. Moreover, the collected temperature, pressure, and salinity signals are dynamically compensated and corrected using prestored model parameters. The dissolved oxygen sensor can transmit signals without distortion and mitigates external interference. Combined with the appropriate detection method, long-term in situ online measurement and detection can be realized accurately and efficiently.

### 3.1. Intelligent Signal Transmission

Intelligent signal transmission refers to the process of amplifying and filtering analog signals to transform them into signals suitable for processing by microprocessors while ensuring no distortion and low loss.

From the point of view of signal processing, dissolved oxygen sensors perceive continuously changing analog signals in the natural environment, which are easily affected by external factors. For example, an electrochemical dissolved oxygen sensor has a high impedance (>1022 Ω), and the weak signals collected are highly susceptible to interference from environmental factors, even when submerged in background noise. Therefore, in signal transmission, it is usually necessary to choose an appropriate high-impedance amplifier circuit [155]. After amplification and filtering, the change ranges of the load current and voltage are reduced, and the extra burr and clutter signals are filtered out. The amplification filter circuit not only effectively amplifies the signal but also amplifies the originally weak signal into a signal with a relatively high amplitude and better signal strength, which improves the accuracy and anti-interference ability of the sensor. Lei designed a high-precision microcurrent measurement circuit and put forward a targeted solution for the key link affecting the accuracy of the microcurrent measurement [156]. He also isolated the microcurrent measurement part and successfully extended the current measurement range of the sensor by several orders of magnitude, which satisfied the accuracy requirement of the sensor. Qi et al. designed a dissolved oxygen sensor with a two-stage amplification and filtering circuit [157]. Firstly, a high-resistance amplifier was used to couple the high-resistance electrode, and then a double-stage amplification circuit was used in a wide range of linear amplifications. After the weak output current of 0–100 nA was filtered and amplified, the signal received by the acquisition circuit was 0.625–2.625 V. Zheng et al. constructed a new high-precision polarographic dissolved oxygen sensor with a low power consumption and high stability performance with an NEC MCU as the core processor [158]. Through the CA3140 I/V circuit, the current signal was first converted into a voltage signal, then amplified by the AD627 precision operational amplifier instrument and finally entered the analog-to-digital (A/D) converter, which greatly reduced the equipment cost and reduced the circuit area.

The analog signal processing technology of optical intelligent dissolved oxygen sensors is also mature. According to the process of signal conversion, optical signals are converted into electrical signals by photoelectric detection elements. However, both the directly obtained optical signals and the converted electrical signals cannot be directly sent to the microprocessor for processing because the signals are weak. Therefore, analog signal processing is still required for the signals before the analog-to-digital conversion. Yang designed a dissolved oxygen sensor based on the fluorescence quenching principle, including a low-noise photoelectric detection circuit with an AD795 as the core and a low-noise amplification and filtering circuit with an OP07 as the core [159]. The hardware detection circuit is sensitive to weak signals with good accuracy and stability. Zhang established a dissolved oxygen sensor based on the fluorescence quenching principle, examined the circuit noise model, and finally selected the OPA376 precision operational amplifier chip as the core element of the alternating current (AC) filter amplification circuit [160]. The signal of the designed circuit has a good SNR and high anti-interference ability. The less amplifier circuit noise there is, the more accurate the measurement result will be. Liao designed a two-stage amplifier circuit using an AD797 as the preamplifier to amplify weak fluorescence reflection signals [161]. A decoupling capacitor was configured in the circuit to suppress noise, and a feedback capacitor acted as a high-frequency filter; thus, the circuit was relatively stable. Gao created an online dissolved oxygen acquisition terminal based on the 32-bit S3C2410X microprocessor and ARM920T kernel [162]. The circuit amplifies and filters the return signal from the sensor and converts the output mA-level current signal into a voltage above 3.3 V. 

According to the Stern–Volmer equation, considering the fluorescence lifetime, Chu proposed an optical oxygen sensor that used simple signal processing based on phase detection [163]. The portable optical oxygen sensor had a 26° phase shift in the 0–100% oxygen concentration range and did not require an original optical filter. Chen proposed that the locking amplifier use a dual-channel orthogonal signal as a reference for constructing the *xy*-coordinate system. The experimental results showed that, in the oxygen-sensing experiment, a 0.058% oxygen concentration caused the phase-locked amplifier to accurately detect a phase shift of 0.1° [164]. Fu proposed a modulated time interval-based complementary metal–oxide–semiconductor (CMOS) sensor chip with multicycle charge modulation for both intensity detection and lifetime extraction in the luminescence-sensing of weak-luminescence sensor devices [165]. He demonstrated the accurate measurement of the oxygen-sensitive chromophore lifetime with sensitivity to oxygen concentrations of 7.5%/ppm and 6%/ppm in both the intensity and lifetime domains. Huang developed a fiber-optic oxygen sensor based on the fluorescence quenching principle, whereby weak fluorescence signals are detected by the phase-locked amplification technique [166].

### 3.2. Digital Signal Processing

After the intelligent signal transmission preprocessing, the signal can enter the microprocessor for further processing through analog-to-digital conversion. However, although high-gain photoelectric conversion detection elements are used in the analog signal processing stage, only low-amplitude signals can be obtained, and the signals also contain interference noise from different sources. Traditional analog signal processing has a low speed and low resolution, and easily transmits distortion, which make it difficult to meet the requirements of real-time and accurate detection of intelligent dissolved oxygen sensors. Compared with analog signals, digital signals have a high anti-interference ability, no noise accumulation, and are convenient for storage and exchange. For integrated equipment, a digital signal is conducive to equipment miniaturization and improves the performance of an intelligent sensor as a whole. Therefore, digital signal processing is required by intelligent sensors because of its incomparable advantages.

Wu adopted digital signal processing technology, namely, the finite impulse response (FIR) digital filter, to filter weak fluorescence signals in the detection system and remove high-frequency noise [167]. Lo proposed a fluorescence sensor using an optical fiber as the conducting material. The system used digital signal processing technology to modulate the LED in the light source part and a digital signal processing chip to enhance the measurement of the fluorescence signal, reducing the bending loss in the optical fiber transmission process [168]. A 256-point complex fast Fourier transform (FFT) was used to convert the time-domain data into the frequency domain by the digital signal processor (DSP) chip, and digital signal averaging and FFT filtering were used to improve the SNR of the sensor and significantly reduce the background noise. The capability of the system to alleviate fiber bend loss was demonstrated.

In addition to improving the SNR and ensuring the transmission quality, the optical dissolved oxygen sensor can also use digital signal processing to calculate the dissolved oxygen concentration. Xu proposed a time-domain fluorescence lifetime-based method for dissolved oxygen detection [169]. Fluorescence was generated by periodic pulsed light excitation with a lifetime that was much longer than the fluorescence lifetime, fully ensuring that the fluorescence generated by the previous excitation completely died out before the fluorescence was generated by the next excitation. Compared with the HQ30d dissolved oxygen analyzer, the measurement error of the dissolved oxygen mass concentration within the range of 0–20 mg/L is less than 0.5 mg/L, and the linear correlation coefficient is up to 0.9992, thereby meeting the requirements of rapid on-site detection. When Stehning measured the phase shift in the frequency domain, he mentioned the FFT algorithm to sample the sinusoidal excitation and response signals and convert them into the frequency domain [170]. The phase shift and measured luminescence attenuation time can be calculated according to the real and imaginary parts of the complex transfer function. The sensor system designed by Zhao is controlled by DSP and uses red and blue lights at different times, allowing the reference light and fluorescence signals to be obtained at different times [171]. The phase-locked loop system is designed to detect the fluorescence lifetime, and the phase difference is solved by the FFT to obtain the dissolved oxygen concentration. Guo turned fluorescent signals into rectangular wave signals by means of a rectifier circuit, and the fluorescence lifetime information was contained in the duty cycle of the rectangular wave [172]. The peak detection circuit obtained the peak value of the real-time fluorescence signal, and the MCU calculated the comparator threshold voltage. A digital phase detector suitable for fluorescence detection was obtained by using a gate circuit and ingenious design, and the phase difference between the fluorescence signal and the falling edge of the reference signal was measured. Tai et al. reported a new circuit for measuring the fluorescence lifetime, which used a square wave to modulate the LED light source pulse signal for fluorescence stimulation and compared the fluorescence signal with the excitation signal to obtain the pulse width of the modulation signal [173]. The signal had a direct triangulation with the fluorescence lifetime and quenching molecule concentration. The design was optimized in every performance of the sensor. This is especially true for the accuracy and speed of actual requirements. It was found that the tangent value of the dissolved oxygen concentration and phase is approximately linear. Feng et al. described a new dissolved oxygen sensor using the optical method, with an emitting diode modulated at 5 kHz as the light source and a red LED as the reference source, to correct the phase offset [174]. The discrete Fourier transform(DFT) algorithm was used to calculate the phase of the photoelectric input signal linearly related to the oxygen concentration. The sensor had a fast response and was suitable for real-time on-site monitoring. Zhang recommended a phase detection method for the AC analog amplification channel in precision photoelectric detection and adopted the digital phase detection method and the three-order integral average method to detect the phase offset [175]. The experimental results showed that the system has good stability, a high detection accuracy, and a quick response (within 40 s), and it can resist all types of interference. The in situ dissolved oxygen monitoring system is suitable for the long-term monitoring of complex marine environments.

**Figure 5:**
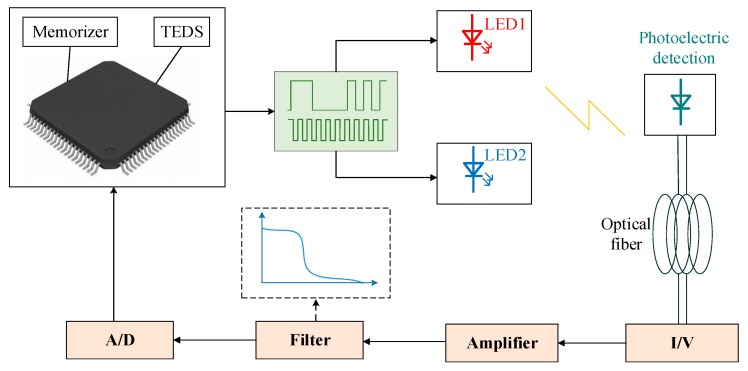
Signal processing module based on the fluorescence lifetime detection principle.

Digital signal processing and analog signal processing cannot be separated, and the intelligent dissolved oxygen sensor has to combine the two signal processing technologies. Figure 5 shows the signal processing module of the fluorescence quenching dissolved oxygen sensor combined with analog signal processing technology and digital signal processing technology. In practical production, analog signal processing technology is often combined with digital signal processing technology. Xu designed a method to detect dissolved oxygen, which uses a microprocessor to control the LED to generate pulsed light signals to excite the fluorescence emission, which is amplified by an amplification circuit, and finally calculates the fluorescence lifetime by comparing the fluorescence intensity of two points to obtain the concentration of dissolved oxygen. This method integrates analog signal processing technology and digital signal processing technology, and has excellent detection performance [169]. Shu used the ATmega128 microcontroller as the core to design the hardware circuit of a working system, including the design of a polarization voltage generation circuit, signal acquisition circuit, temperature compensation circuit, and serial communication circuit [176]. This digital microscale dissolved oxygen analyzer met the μg/L-level precision requirement. Yin established an intelligent optical dissolved oxygen sensor with the STM32F103 as the core controller, which converted fluorescence signals into electrical signals, and then the continuous electrical signals were transformed into digital signals that could be processed by the MCU through A/D conversion [177]. Moreover, an intelligent and networked sensor was realized through Transmission Control Protocol(TCP) configuration, with a stable sensor performance and high measurement accuracy. The combination of analog signal processing technology and digital signal processing technology enables the intelligent dissolved oxygen sensor to achieve better results.

### 3.3. Adaptive Real-Time Compensation Correction

In the liquid phase, the dissolved oxygen sensor is easily affected by the temperature, pressure, salinity, and other factors, which cause large deviations in the measurement results. In the research on dissolved oxygen sensors manufactured by Aanderaa and Sea-Bird, the concerns of temperature, salinity, and pressure were also explicitly raised [178,179]. Therefore, intelligent dissolved oxygen sensors must deal with these influencing parameters. The traditional dissolved oxygen sensor often adopts the method of hardware compensation. For example, the dissolved oxygen sensor dynamically compensates for the temperature by a thermistor in series. Zhang installed thermosensitive elements in the circuit to automatically compensate for temperature changes to prevent the output current from changing as a result [180]. However, the traditional sensors have shortcomings, such as calibration difficulties and poor interchangeability. To accurately detect the concentration of dissolved oxygen, in the design of sensors, data correction, the compensation and correction of influencing factors, and automatic sensor calibration are achieved by both hardware and software [156,181,182]. Based on the idea of multisource information fusion, Zhao et al. studied the method of the correction and compensation of multisource dissolved oxygen information and developed a rapid detection field instrument for dissolved oxygen with an ultralow power consumption and an MSP430F427 as the processor [183]. The sensor can be widely used for the rapid detection of dissolved oxygen in the field, but its error is too large for use in aquaculture environments; thus, the sensor hardware needs to be improved.

The water temperature has the most notable effect on the dissolved oxygen concentration. The specific technology that makes the technical index and performance of the sensor resistant to temperature changes is called temperature compensation technology. The temperature signals detected by the dissolved oxygen sensor can be used for the adaptive real-time compensation and correction of the dissolved oxygen using a preset temperature compensation and correction model in the microprocessor.

Zheng connected a temperature sensor with a single-chip microcomputer, and a temperature correction algorithm was automatically installed in the sensor to ensure the output accuracy [158]. Zhang et al. designed a software calibration method for dissolved oxygen electrodes according to the need for temperature compensation in dissolved oxygen measurement [184]. Under the condition that the dissolved oxygen transmitter works in the linear region, the temperature compensation of the dissolved oxygen measurement was realized by using the data obtained in the software calibration process. The experiments showed that the measurement results obtained by the sensor using the compensation strategy above are similar to those obtained by Mettler’s dissolved oxygen transmitter 4500. This method not only ensures the accuracy of the dissolved oxygen measurement but also greatly reduces the hardware cost. Huang et al. adopted a coated polarographic dissolved oxygen sensor, which made the negative temperature coefficient thermistor used for temperature compensation in the traditional mode obsolete and replaced it with the high-precision digital temperature sensor DS18B20 produced by the DALLAS company in the United States [185]. The hardware temperature compensation was improved using software compensation. The temperature compensation range is between 0 and45 °C, and the measurement accuracy is 0.1 mg/L, with a response time of less than 10 s. In their design of the polarographic electrode, Zhang used the software temperature compensation method to eliminate the temperature influence on the dissolved oxygen measurements by injecting oxygen into water at different temperatures until oxygen saturation occurred, and the output current of the dissolved electrode was then measured [186]. The temperature coefficient K was calculated from the measurement results, which can be used to correct the measurement results. This method is more accurate than the traditional hardware temperature compensation. Xin simultaneously measured the same saturated oxygen solution with/without the temperature compensation of the sensor using the two-electrode dissolved oxygen sensor produced by Orbisphere [187]. The results showed that the sensor with temperature compensation has good detection and tracking effects of the dissolved oxygen and a high measurement accuracy, and the detection results are closer to the real values.

In the optical dissolved oxygen sensor, both the fluorescence-sensing film and the light source are highly dependent on the temperature. The adaptive compensation of the optical temperature is of great significance for the accurate measurement of the dissolved oxygen concentration. Before analyzing the sample, San-Shan activated an external interruption mechanism to detect the ambient temperature and conduct temperature compensation, which eliminated the temperature influence on the fiber optic gas sensor to a certain extent [188]. Palma analyzed an empirical calibration function of a dissolved oxygen sensor based on the fluorescence quenching principle and applied it successfully to a sensor he designed by himself. The experiment compensates for the effect of the temperature on the fluorescence quenching process. The measurement results were compared with those of the laboratory method, and the overall deviation was not more than 0.35% [189]. Weihong obtained the functional relationship between the fluorescence lifetime value, fluorescence quenching coefficient, and water temperature through experimental analysis and proposed temperature compensation through a function model [171]. After the calibration, digital filter processing was carried out to filter out random pulse interferences and improve the smoothness of the measured data. Finally, the measurement accuracy of the test prototype was 0.1 mg/L. Based on the design idea of the IEEE1451 intelligent sensor, Ding compiled software to complete the self-calibration of the sensor and carried out the adaptive compensation and correction of the temperature through curve-fitting [190]. The measurement results showed good measurement accuracy. Li et al. established a temperature-based online monitoring model [191]. The original signal can be converted into a correction signal according to the calibration parameters stored in the transducer electronic data sheet (TEDS), and temperature compensation can be carried out by the curve-fitting method. When using the calibration parameters stored in the TEDS, the sensor can directly determine the accurate value, which not only facilitates automatic identification but also solves the problem of the automatic correction of the sensor and truly realizes intelligent compensation and correction.

In addition to the usual temperature compensation, the air pressure compensation is generally considered. According to Henry’s law, the gas solubility is proportional to its partial pressure. The partial oxygen pressure is related to the altitude of the region, and the difference between the plateau and plain areas can be up to 20%; thus, the dissolved oxygen sensor must be compensated according to the local atmospheric pressure before use. Some instruments are equipped with a barometer inside and can be automatically calibrated; other instruments are not equipped with a barometer and should be calibrated according to data provided by local weather stations. Incorrect settings lead to a large measurement error. Bin proposed pressure compensation [192]. To verify the technical indicators of the designed dissolved oxygen sensor, the sensor measurement results were compared with the dissolved oxygen data in water measured by iodimetry under the same water sample and test environment conditions, and the sensor accuracy error was 0.07 mg/L. Ding converted the compensation algorithm into the standard form of IEEE1451.2 and then stored the correction parameters in the TEDS to realize the temperature and pressure compensation of the dissolved oxygen intelligent sensor [193]. The sensor automatically corrected the transmitter output according to the current temperature and atmospheric pressure and saved the slope calibration parameter to the calibration TEDS of the Smart Transducer Interface Module(STIM). The calibration result was compared with that of the HACH water analyzer, and the output error was less than 1%.

The solubility of oxygen in water decreases with the increasing salinity, and salinity compensation must be performed. When the salinity (expressed as total salt) is below 35 g/L, the above relationship can be considered linear. Langdon designed a pulse potential electrode to improve the instrument performance [194]. Software was used to precisely correct the sensor output for temperature dependence using the activation energy of permeation and the Arrhenius equation. The activity coefficient, which depends on the temperature and salinity, is computed to correct for the salinity. Li also proposed pressure and salinity compensation and provided a curve-fitting analysis method [191]. The compensation parameters were stored in the TEDS, and the compensation effect was good. This compensation met the requirement of an intelligent optical dissolved oxygen sensor. Table 5 compares the performance of the proposed adaptive dissolved oxygen sensors.

Some sensors do not distinguish influencing factors but instead directly carry out an overall correction [195]. Hahn designed a self-diagnostic dissolved oxygen sensor, which can automatically correct dissolved oxygen measurement values and diagnose and analyze membrane pollution problems such as algae, sediment, or suspension droplets, to realize the long-term in situ online detection of the dissolved oxygen concentration without too frequent inspections of the sensor state [196]. Eminaga reported an approach to enable the continuous long-term operation of such sensors by in situ self-calibration [197]. Sensor calibration often requires the detection of the system’s complete characteristic line, that is, the current output as a function of the oxygen concentration. In addition to the above factors, intelligent sensors can also be used to compensate and correct for other factors [198].

The key technologies of intelligent dissolved oxygen sensors include intelligent signal transformation, digital signal processing, and adaptive real-time compensation and correction. In terms of intelligent signal transformation and transmission, both electrochemical and optical dissolved oxygen sensors require the analog signal processing of the collected continuous signals; weak signals are amplified, and high-frequency and other interference signals are filtered to facilitate signal processing by the processor without signal loss. When the signal enters the microprocessor, it is necessary to consider the digital signal processing technology. The processed digital signal has a high anti-interference ability and no noise accumulation, which is more conducive to the miniaturization of the integrated equipment and improves the performance of the intelligent sensor as a whole. In addition, the temperature, pressure, salinity, and other external factors have an impact on the test results. Although the traditional hardware compensation method can reduce this effect to a certain extent, the method has a low compensation accuracy when the external environment changes dynamically. Adaptive real-time compensation is achieved by combining software and hardware. The model parameters preexisting in memory or TEDs can be accessed in real time, and the dissolved oxygen concentration value can be calculated by a specific model. The intelligent technology of dissolved oxygen sensors not only facilitates the signal processing of the sensor but also truly solves the problems of self-correction and self-diagnosis and, thus, improves the accuracy and precision of measurement.

At present, the intelligent dissolved oxygen sensor occupies a large market. Some well-known manufacturers of dissolved oxygen sensors and their products are listed in Table 6. Basically, these companies made some achievements in sensor intelligence. For example, Mettler’s optical dissolved oxygen sensor can be “plug and play”, with a built-in microprocessing chip, compatible with digital and analog signals, and intelligent functions such as automatic calibration, automatic temperature compensation, and self-diagnosis information. ProOBOD series optical measuring instrument manufacted by YSI company is equipped with a high-performance pre-amplifier to ensure long-distance transmission without interference, and its digital dissolved oxygen sensor stores calibration data without recalibration. Aanderaa and Sea-Bird spend a lot of effort on compensation calibration, and their dissolved oxygen sensors are highly intelligent and have good detection results [199]. Therefore, experts often take the dissolved oxygen sensors of Aanderaa and Sea-Bird as the basic equipment for research on intelligent dissolved oxygen sensors [200]. Several of the sensors in Table 6 now have automatic temperature compensation, but most rely on the artificial input of salinity and pressure values to compensate for dissolved oxygen. Among them, the most advanced compensation technology is to obtain temperature, pressure, and conductivity (salinity) in real time through multi-parameter sensors, and then the built-in microprocessor can call the pre-stored calibration or compensation function to calculate a relatively accurate value of dissolved oxygen concentration. Therefore, dissolved oxygen sensor technology still needs to be further developed. In addition, it can be found from the principle of these companies’ products that intelligent technology tends to use the optical principle of dissolved oxygen sensors.

## 4. Conclusions

The dissolved oxygen is an important evaluation index of the water quality, which is applied in many fields, such as environmental monitoring, aquaculture and medical-grade food. Particularly in aquaculture, the dissolved oxygen is the key premise for the healthy growth of aquatic organisms. Therefore, it is important to measure the concentration of dissolved oxygen accurately and in real time.

Dissolved oxygen detection technology developed from iodometric titration was initially used as a laboratory method. Due to the development of electrochemical technology and fluorescence technology, electrochemical and optical dissolved oxygen sensors were designed according to their respective principles. At present, the commonly used methods of dissolved oxygen detection include iodometric titration, electrochemical detection, and optical detection. Iodometric titration is a purely analytical chemical method with high accuracy and good repeatability but complicated operation, and the method also consumes oxygen. The method is only suitable for laboratory analysis. Polarography is currently the most widely used electrochemical method, with a simple structure, wide range of applications, and mature technology. At the same time, polarography also has limitations, such as a long polarization time, water flow rate requirement, oxygen consumption of the process, and difficulty in maintenance. Although the online measurement of the dissolved oxygen concentration can be achieved, the dissolved oxygen concentration cannot be measured continuously for a long time due to the need for regular maintenance. The fluorescence method overcomes the problem of oxygen consumption in both the titration and electrochemical methods and has a high sensitivity and fast reaction time. The fluorescence quenching dissolved oxygen sensor based on optical-fiber technology has a high anti-EMI ability and does not require frequent maintenance, making it more conducive to the requirements of long-term in situ measurement. Thereafter, research on electrochemical detection technology mainly focused on electrode materials, and the development of optical sensors mainly includes the improvement of new sensitive materials and matrixes. Material improvement resulted in enhanced sensor performance to a certain extent, but it is still difficult to solve the problems caused by external interferences, and intelligent sensor technology can compensate for the shortcomings of traditional detection technology. Intelligent dissolved oxygen sensors process dissolved oxygen signals from three aspects, intelligent signal transmission, digital signal processing, and adaptive real-time compensation and correction, and they can obtain accurate dissolved oxygen measurements in real time.

The development direction of dissolved oxygen sensors is clear. Firstly, new materials will continue to be examined. New materials can solve the limitations of dissolved oxygen sensors directly. For example, new materials will directly improve the sensor performance (long life, low loss, and high sensitivity), without requiring the frequent maintenance, calibration, and cleaning of the sensor, which greatly reduces the staff workload. Secondly, in terms of the sensor type, the optical dissolved oxygen sensor based on the fluorescence quenching principle, with its advantages of less maintenance, no warm-up time, and little calibration drift, will become the dominant dissolved oxygen sensor market in the future, and its market share will surpass that of electrochemical dissolved oxygen sensors. Thirdly, optical fibers will appear more frequently in the design of optical dissolved oxygen sensors, which are convenient for their remote transmission and high anti-interference abilities and can readily achieve in situ dissolved oxygen measurement in oceans and large lakes. Fourthly, most importantly, intelligent dissolved oxygen sensor technology will become the focus of future research and mainstream development. The intelligent dissolved oxygen sensor can intelligently process signals by using analog and digital signal processing technologies and performing real-time dynamic compensation and correction for the temperature, pressure, salinity, and other interference factors, thereby greatly reducing the manual operation and management cost and directly or indirectly reducing human errors. In summary, it is not difficult to realize that intelligent fiber-optic dissolved oxygen sensors based on the fluorescence quenching principle are suitable for remote online measurement, without the need for frequent maintenance and a high anti-interference ability, which meets the accuracy and real-time requirements of dissolved oxygen measurement in various application scenarios. This type of sensor, however, still has technical issues and challenges, which will be the focus of future research.

## Figures and Tables

**Table 1:** Comparison of polarographic electrochemical dissolved oxygen sensors.

Electrode	Coefficient of Correlation (Determination)	Sensitivity	Linear Response Range	Detection Limit	Response Time	Reference
βCDSH ^1^+ FeTMPyP ^2^ + CDAuNP ^3^	*r* = 0.999	5.5 μA·L·mg^−1^	0.2–6.5 mg·L^−1^	0.02 mg·L^−1^	-	[54]
Three electrodes: nickel–salen + platinum, SCE ^4^, platinum	*r* = 0.9976	-	3.95–9.2 mg·L^−1^	0.71 mg·L^−1^	-	[55]
Three electrodes: hemin, Ag, AgCl, platinum wire	*R^2^* = 0.9872	8.5 μA∙L∙mg^−1^ (20.7μA·cm^−2^)	2–7 mg·L^−1^	-	200 s	[56]
Three electrodes: F-BDD ^5^ + boron-doped CVD ^6^, Ag, AgCl, Pt	*R^2^* = 0.9988	0.1422 ± 0.006 nA·μM^−1^	0–273.75 μM	0.63 μM	0.01 s	[57]
Three electrodes: RGO-Ag ^7^ + GCE ^8^, Ag, AgCl, platinum wire	*R^2^* = 0.991	0.205 μA·μM^−1^	1–120 μM	0.031 μM	<5 s	[59]
Three electrodes: RuO_2_, AgPd, Ag, AgCl	*R^2^* = 0.9694	0.560 μA L·mg^−1^	3–11.7 mg·L^−1^	-	4 min	[61]
Three electrodes: nickel–salen + platinum, SCE, platinum	-	-	3.95–9.20 mg·L^−1^	0.17 mg·L^−1^	-	[62]

^1^ Mono-(6-deoxy-6-mercapto)-β-cyclodextrin (βCDSH). ^2^ Iron(III) tetra-(*N*-methyl-4-pyridyl)-porphyrin (FeTMPyP). ^3^ Cyclodextrin-functionalized gold nanoparticles (CDAuNP). ^4^ Saturated calomel electrode (SCE). ^5^ Boron-doped diamond microelectrodes modified by CF4 plasma (F-BDD). ^6^ Chemical vapor deposition (CVD). ^7^ Reduced graphene oxide–silver nanoparticles (RGO-Ag). ^8^ Glassy carbon electrode (GCE). Here, *r* is the coefficient of correlation, indicating the degree of linear correlation between dissolved oxygen concentration and current intensity. *R^2^* is the coefficient of determination, indicating the regression fitting degree of the curve of dissolved oxygen concentration and current intensity.

**Table 2:** Comparison of polarographic and galvanic dissolved oxygen sensors.

	Polarographic Sensor	Galvanic Cell-Type Sensor
**Precision**	High	Lower
**Technology**	More mature	Mature
**Response time**	Long polarization time (approximately 5 to 15 min)	Short response time
**Service life**	Long	Short (the lifetime is related to the electrode materials and redox reactions)
**External circuit**	Polarography requires external circuits.	The galvanic method does not require external circuits.
**The intensity of the output**	Stronger current than the galvanic-type sensor	The current is low (generally at the μA level)
**Applications**	Polarographic sensors are troublesome to use outdoors	The galvanic sensor is suitable for outdoor use without an external circuit
**Common issues**	These sensors all experience disturbances by chlorine, sulfur dioxide, iodine, bromine, electromagnetic interference, etc.	

**Table 3:** Comparison of fluorescence quenching dissolved oxygen sensors.

Oxygen Indicator	Matrix	Emission Wavelength	Optical Fiber (yes/no, Y/N)	Sensitivity	Response Time	Reference
PtOEP ^1^	PEMA ^2^	645 nm	Y	15 (I_N2_/I_O2_ -1)	41 ms	[110]
Chlorophyll–zinc complex	Silica gel	640/680 nm	N	-	-	[111]
[Ru(dpp)_3_][(4-Clph)_4_B]_2_^3^	Silica gel	604 nm	N	3.6 ppm (I_0_/I -1)	<1 s	[113]
PtOEP	PMMA	-	Y	T_0_/T -1 = 1.75	<0.8 s	[115]
PtOEP	PMMA	647 nm	Y	K_SV_ = 0.022	-	[116]
	PEMA			K_SV_ = 0.118	-	
	PPMA ^4^			K_SV_ = 0.195	<100 ms	
[Ru(dpp)_3_]Cl_2_	Sol–gel	603 nm	Y	I_0_/I = 3.6	200 ms	[117]
Ru(bpy)_3_Cl_2_	Silica–Ni–P composite	603 nm	N	I_0_/I_100_ = 2.49	300 s	[118]
PtTFPP ^5^ and dye-entrapped core–shell silica nanoparticles	TEOS ^6^/C_8_ TEOS	650 nm	Y	I_0_/I = 117 (0–40 mg/L)	694 s	[119]
Ru(dpp)_3_^2+^	TMOS ^7^/DiMe-DMOS ^8^	592 nm	N	I_0_/I = 16 (0–100%)	100 s	[120]
PdTFPP	Octyl-triEOS/TEOS sol-gel	643 nm	Y	0.0554 (40 °C)	11 s	[127]
PtOEP		676 nm		0.12 (40 °C)	10 s	
Ru(dpp)_3_^2+^		590 nm		0.0015 (40 °C)	10 s	

^1^ Luminophore-platinum-octaethyl-porphyrin (PtOEP). ^2^ Poly(ethyl methacrylate) (PEMA). ^3^ Tris(4,7-diphenyl-1, 10- phenanthroline)ruthenium(II) ditetrakis(4-chlorophenyl)borate ([Ru(dpp)3][(4-Clph)4B]2). ^4^ Poly(methyl methacrylate) (PMMA); poly(ethyl methacrylate) (PEMA); poly(propyl methacrylate) (PPMA). ^5^ 5,10,15,20-tetrakis (pentafluorophenyl) 21*H*, 23*H*-porphine palladium(II) (PdTFPP). ^6^ Tetraethylorthosilicate (TEOS). ^7^ Tetramethoxysilane (TMOS). ^8^ Dimethyldimethoxysilane (DiMe-DMOS).

**Table 4:** Comparison of three methods for the determination of dissolved oxygen.

	Winkler Method	Clark Method	Fluorescence Quenching Method
**Precision**	As a benchmark method, the Winkler method has the highest detection accuracy.	The detection accuracy is good but is easily affected by water quality and electromagnetic interference.	The fluorescence quenching method is hardly affected by the water quality and has a high anti-electromagnetic interference ability; thus, the detection accuracy is high.
**Response time**	The Winkler method is a laboratory method that is complex and takes the longest time.	The Clark method requires the polarization of the electrode (approximately 5 to 15 min), so the response time is long.	The fluorescence quenching method has the fastest response time (up to the ms level).
**Oxygen consumption**	The titration process consumes oxygen.	The redox reaction at the electrode consumes oxygen.	The fluorescence quenching process is reversible and does not consume oxygen.
**Remote sensing**	The Winkler method cannot easily achieve remote measurement and, usually, water samples must be analyzed in the laboratory.	The Clark method can achieve remote detection, but the signal transmission will be distorted; thus, the detection results are not accurate.	The fluorescence quenching method can use an optical fiber to transmit signals, with a low signal loss and long transmission distance, and can achieve remote detection (optical fibers can be up to 20 m long).
**Maintenance**	No	The sensor-based Clark method requires frequent maintenance.	The sensor-based fluorescence quenching method does not require constant maintenance.
**Interference factors**	Water turbidity, nitrite, iron ions, free chlorine, etc.	H_2_S, SO_2_, pH, electromagnetic interference, etc.	Cl_2_, etc.
**Application**	Laboratory and fewer samples.	Agriculture, forestry and fishing, biological medicine, etc.	Agriculture, forestry and fishing, life sciences, strong electromagnetic interference, and other harsh environments.
**Market share**	-	Low cost, wide application.	Large market share and high demand.

**Table 5:** Comparison of adaptive dynamic compensation correction sensors.

Hardware	Software			Deviation (Precision)	Detection Range	Response Time	Reference
	T ^1^	S ^2^	P ^3^				
Y	Y	Y	N	±0.2 mg/L	0–20 mg/L (0–40 °C)	-	[156]
Y	Y	Y	Y	≤±5%	0–15 mg/L (0–60 °C)	-	[183]
Y	Y	N	N	≤±5%	-	-	[184]
Y	Y	Y	Y	0.1 mg/L	0–20 mg/L (0–45 °C)	<10 s	[185]
Y	Y	N	N	<1 μg/L (<3%)	-	<120 s	[58]
Y	Y	N	N	±0.1 mg/L (0.5%)	0–20 mg/L	-	[158]
Y	Y	Y	Y	Relative standard deviation (RSD) <2% (0.01 mg/L)	0–20 mg/L	<3 min	[191]
Y	Y	Y	Y	±0.07 mg/L	0–20 mg/L	-	[192]
Y	Y	Y	Y	<1%	0–20 mg/L	-	[193]

^1^ Temperature. ^2^ Salinity. ^3^ Pressure.

**Table 6:** Intelligent dissolved oxygen sensor products.

Company	Product	Principle	Temperature Compensation	Salinity Compensation	Pressure Compensation	Accuracy	Response Time
Aanderaa	4835	luminescence quenching	The sensor has a thermistor to realize automatic temperature compensation.	Salinity compensation is required when salinity changes are >1 mS/cm.	Salinity compensation is required for pressure >100 m.	<8 µM	<10 s
	4831/4831F					<2 µM	<2s
	4531					<8 µM	<2 s
WTW	TriOxmatic 700 IQ	Polarographic	The sensor has a built-in NTC ^1^ to realize automatic temperature compensation (−5 to 60 °C).	Manually set compensation for 0–70 ppt.	Automatic compensation.	±0.1 mg/L	180 s
	TriOxmatic 702 IQ					±0.01 mg/L	30 s
	FDO 700 IQ	Luminescence quenching	The sensor has a built-in NTC to realize automatic temperature compensation (−5 to 50 °C).	When the salinity is >0.1%, salinity compensation is carried out.		±0.05 mg/L	<150 s
	FDO 701 IQ					±0.1 mg/L	< 60 s
Mettler	SG9	Fluorescence quenching	The sensor has a built-in NTC to realize automatic temperature compensation.	When the salinity is >1 ppt, manually input the salinity value and automatically compensate for the salinity (0–42 ppt).	The sensor has a barometer to automatically or manually compensate for atmospheric pressure.	±0.1 mg/L	-
	SG6					±0.5% mg/L	90 s
HACH	HACH LDOTM HQ10	Fluorescence quenching	Sensor with 30 kΩ thermistor for automatic compensation (0–50 °C).	Automatic compensation (0–70‰).	Automatic compensation (400–1100 mBar).	±0.1 mg/L	< 30 s
	HQ30d		The sensor has a built-in NTC to realize automatic temperature compensation.	The salinity measured by the conductivity electrode is automatically compensated.	Automatic pressure compensation.	-	-
Sea-Bird Scientific	SBE 43	Polarography	Due to the serious drift caused by dirt pollution, the temperature, salinity, and air pressure are compensated by the calibration formula.			-	-
	SBE 63	Fluorescence quenching	Each SBE 63 is calibrated individually in a temperature-controlled bath.	Salinity and pressure impacts on sensor response are each checked at two separate points.		0.1 mg/L	<6 s
YSI	EcoSense ODO200	Fluorescence quenching	Automatic temperature compensation.	Manually enter the salinity value to compensate	Manually input the pressure value to compensate (contained barometer)	±0.15 mg/L	-
	Pro20	Electrochemistry principle	All cable assemblies have built-in temperature sensors.	Manually set compensation for 0–70 ppt.	Automatic barometric pressure compensation.	±0.2 mg/L	8 s
	ProSolo ODO	Fluorescence quenching	Built-in thermistors for temperature compensation (−5 to 50 °C)	Manually input the salinity value; the sensor allows real-time salinity compensation.	The sensor has a barometer.	±0.1 mg/L (0–20 mg/L)	-
Kongsberg	CONTROS HydroFlash® O2	Fluorescence quenching	-	-	-	±1%	<3 s
OxyGuard	Handy Polaris	Galvanic type	Self-temperature compensation.	Set the salinity value manually for automatic compensation (0–59 ppt).	-	±1%	<20 s

^1^ NTC: negative temperature coefficient.
